# Editorial: Insights in biomaterials 2022 / 2023—novel developments, current challenges and future perspectives

**DOI:** 10.3389/fbioe.2024.1364724

**Published:** 2024-01-23

**Authors:** Hasan Uludağ, Yunbing Wang, Nihal Engin Vrana, Candan Tamerler, Chandra Kothapalli, Milana C. Vasudev

**Affiliations:** ^1^ Faculty of Engineering, University of Alberta, Edmonton, AB, Canada; ^2^ National Engineering Research Center for Biomaterials, Sichuan University, Chengdu, China; ^3^ Spartha Medical, Strasbourg, France; ^4^ Mechanical Engineering, University of Kansas, Lawrence, KS, United States; ^5^ Washkewicz College of Engineering, Cleveland State University, Cleveland, OH, United States; ^6^ Bioengineering, University of Massachusetts Dartmouth, North Dartmouth, MA, United States

**Keywords:** biomaterials, scaffold, tissue engineering, therapy, diagnostics, drug delivery, biocomatability

We are now entering the third decade of the 21st century, and, especially in the last few years, the achievements made by scientists have been exceptional, leading to major advancements in the rapidly growing fields of bioengineering and biotechnology. New biomaterials designed with advanced features have been the driving force behind the applications in bioengineering and biotechnology fields. This annual Research Topic, which highlights article submissions typically from our Editorial Board members, looks to explore new insights, novel developments, current challenges, latest discoveries, recent advances, and future perspectives in the field of ***Biomaterials***. This Research Topic solicited brief, forward-looking contributions that describe the state of the art, outlining recent developments and major accomplishments that have been achieved and that need to occur to move the field forward. We wanted to shed light on the progress made in the past decade in the biomaterials field, and on its future challenges, to provide a thorough overview of the field. This article Research Topic is intended to inspire, inform and provide direction and guidance to researchers in the field. Eleven articles have been finally accepted for this Research Topic which were a mixture of review papers and technical articles.

The review paper by Tiemeijer and Tel summarized the current state of affairs in utilization of ***hydrogels*** for single cell microgel production. Hydrogels, with their high-water content and being readily tunable for physicochemical features, offer an opportunity to create suitable microenvironments for cell cultivation at microscale so as to foster selection of individual cells (or clones) for selection for a variety of reasons. While microdrop production has been used for cell encapsulation since 1980s, using microfluidics devices that emerged in the last decade provides exquisite control over the hydrogel features at the micro scale. Common materials used for microgel production was reviewed as well as a survey of applications including sequencing, biosensing using entrapped cells and enhanced cultivation for various purposes, including therapy. The technology is bound to yield novel diagnostic and therapeutic approaches based on cellular cultivation. The review article by Cassenellas et al. have focussed on recent advances in ***engineering nanotopographic substrates*** for cell studies. As in engineered microgels above, ‘nano-engineered’ surfaces could provide unique and improved features for cultivation of cells intended for a variety of reasons. The authors briefly summarized the mechanistic effects of nano-engineered topography on cellular processes, while providing a summary of main fabrication techniques to achieve the desired nanotopographies. Various biological applications were summarized in this context, and the authors briefly commented on the ‘stimuli-responsive’ nanotopographies to present fruitful opportunities in the future.

The nano-structured particles, as in surfaces, have also found widespread use in medicine. The early host response to such nanoparticles following the initial opsonization is typically the cytokine secretion, which has important implications on host physiology as well as the compatibility of the nanoparticles in the long run. Nasrullah et al. summarized the most recent literature on ***cytokine responses to nanoparticulate systems*** and identified the main factors of nanoparticles affecting this response. For diseases arising from the altered cytokine pathophysiology, attempts to silence the individual components of cytokine response are summarized, and the roles of nanoparticle features in this respect were presented. The authors provide their perspective on the possibility of engineering nanoparticle delivery systems with controlled cytokine responses as the basis of new therapeutic modalities.

In an original article, Cervello et al. probed the ***protease-degradable hydrogels*** with multifunctional biomimetic peptides for bone tissue engineering. Integrin-binding and osteogenic Bone Morphogenetic Protein-2 (BMP-2) derived peptides were used to functionalize hydrogels, leading to successful cultivation of mesenchymal stem cells (MSCs) and significantly improving their osteogenic differentiation. Such biomimetic materials can find application in tissue engineering efforts when constructing functional tissues *ex vivo* as well as in regenerative medicine to stimulate innate bone regeneration *in situ*. The design principles emanating from such exquisitely-engineered biomaterials are bound to be applicable to other scaffolds intended for other types of tissues as well. The perspective article by Jennissen on one of the first morphogens discovered, BMP-2, keeps the light shining on this enigmatic protein; the author describes experimental systems composed of BMP-2 and electrospun poly (L-lactide) nanofibers that point out new mechanisms of action for BMP-2 (pro- and anti-angiogenic functions) at picomolar range, which is substantially lower than typical doses used in clinical setting (mg/mL) and small preclinical animal models (μg/mL). Careful considerations and better understanding of physiochemical features and *in situ* levels of the protein is bound to guide better design of ***devices incorporating tissue-inducing morphogens***, aiming for more physiological tissue repair.

The compiled Research Topic provided valuable perspectives on specific types of biomaterials utilized in biotechnological and medical applications. ***Cellulose***, one of the most abundant materials in nature, is a versatile biomaterial that has been explored for numerous applications to improve human health. Its modification is continually attempted to finetune the physicochemical properties for new applications. Fatema et al. summarized recent developments in modified celluloses, summarizing physical and chemical means to undertake the desired modifications. The range of applications pursued with cellulose are presented with a keen eye on possible future developments. Focus on nanoscale features are particularly emphasized as the next frontier in celluloses’ use as a biomaterial. With respect to a specific biomaterial for a target application, Chen et al. highlighted the current state of three-dimensional printing of ***polyetheretherketone*** (PEEK) and its multifunctional modification for dental implants. PEEK is an attractive polymer that can be engineered to display an elastic modulus close to that of the mineralized tissues. That makes it suitable for implantation at such sites from a mechanical perspective but its other features pose severe barriers for full histocompatibility. The authors reviewed the recent attempts to modify the material and deploy it in 3D-printing processes to create the ideal dental implant. The experience gained by the PEEK could illuminate the path for new generation of synthetic polymers as biomedical implants. In a review article, Noh et al. explored the technology of stimuli-responsive ***elastin-like polypeptides*** (ELPs) for biomedicine and beyond, especially their potential application as programmable soft actuators. A summary of stimuli-response biomaterials has been presented as the foundation of biomedical actuators, driven by electrical, thermal, pH and light responsive elements. The ELPs stand out as thermoresponsive materials; the authors presented a spectrum of uses for ELPs and provided differentiated features of ELPs as compared to other thermoresponsive materials. Recent developments in the field have been summarized in detail while the critical next-steps to move ELP towards actuators has been briefly outlined.

Two articles in this described devices intended for targeted applications*.* Zhang et al. reviewed recent developments in interventional devices for mitral valve repair with implantation of artificial chords. The article focussed on mitral regurgitation and its repair, outlining several approaches to surgical repair and highlighting currently used devices in a clinical setting. The use of one of the first synthetic polymers in modern medicine, namely, expanded ***polytetrafluoroethylene*** (ePTFE), has been highlighted in this application. More importantly, factors contributing to the failure of artificial chords have been probed. This is critical to develop the next-generation materials (or surgical approaches) to improve mitral interventions. The authors highlight the expected evolution of the field in the direction of interventional transcatheter chordae repair devices. On the other hand, Veit et al. report an original study on a versatile, bioengineered skin reconstruction device designed for use in austere environments. ***Silk fibroin with an antioxidant hyaluronic acid*** derivative were formulated into a skin compliant device with controlled drug delivery and self-adherent features. This engineered device performed well in an *in vitro* model with good integration into the tissue by allowing cellular migration into and above the device. The prototype described in this study will illuminate the path for simple-yet-functional regenerative devices where limited medical resources are available.

Finally, a review article by Sun et al. describes the past experience with ***biodegradable films*** for soil by conducting a 30-year systematic review. Polymer science has increasingly provided the foundation and the materials for biodegradable films with minimal environmental footprint. Although the focus is not biomedical use of materials, the analytical methodology on published literature described in this paper as well as the mechanistic insight into biodegradation mechanism(s) may pave ways for development of new materials in biomedical enterprise. The environmental considerations and clinical perspective both recognize the biodegradation as ideal to reduce waste and to leave a minimal footprint in the host. This philosophy could promote ecologically sustainable development, and this article may further sensitize biomedical engineers and scientists in this regard.

The Research Topic of articles provides important additions to biomedical literature where traditional, mechanically oriented and inert biomaterials as well as more recent biomimetic regenerative biomaterials are formulated for various applications. Emphasis on application-oriented contributions emphasize the need to design, manufacture and optimize the biomaterial and device features for the intended applications. Our imagination and a plethora of state-of-the-art tools drive this venture. Transdisciplinary scientific and engineering pursuits are evident in the collected papers where the traditional silos are routinely crossed to yield effective interventions for the benefit of patients.

